# Proportion of parental genomes in hybrids *Allium cepa × A. roylei* determines which one becomes dominant

**DOI:** 10.1002/tpg2.70016

**Published:** 2025-03-28

**Authors:** David Kopecký, Martin Duchoslav, Olga Scholten, Jana Kneřová, Marek Szecówka

**Affiliations:** ^1^ Centre of Plant Structural and Functional Genomics, Institute of Experimental Botany Czech Academy of Sciences Olomouc Czech Republic; ^2^ Department of Botany Palacký University Olomouc Czech Republic; ^3^ Plant Breeding Wageningen University & Research Wageningen The Netherlands

## Abstract

Interspecific hybridization leads to complex interactions between the parental genomes, often in the form of genome dominance, where one genome prevails over the other. This phenomenon has been attributed to differential chromosome behavior during meiotic division and may involve either female or male meiosis, or both. In hybrids of *Allium cepa × A. roylei*, only female meiosis is involved, favoring the transmission of *A. roylei* chromosomes; male meiosis leads to the development of gametes with equal proportion of parental genomes. Female meiotic drive shifts the genome composition from 8R (*A. roylei*) + 8C (*A. cepa*) chromosomes in F_1_ to 9.3R + 6.7C in F_2_. In this study of two successive backcross generations with *A. cepa* (BC_1_ [first backcross generation] and BC_1_F_1_ [progeny after intercross of the first backcross generation]), we observed a change in genome dominance: the *A. roylei* genome, initially dominant during the meiosis in the F_1_ hybrids, became submissive in BC_1_, resulting in a genome composition skewed toward *A. cepa*. Among 23 BC_1_ and 236 BC_1_F_1_ plants, we observed a significant deviating trend of gradual reduction in *A. roylei* chromosome representation. The reduction was higher in the lineages with more unequal starting proportion of the parental genomes. This study highlights the dynamic nature of genomic interactions in hybrids and raises questions about the underlying molecular mechanisms driving these changes in dominance, as well as the potential for manipulating these interactions for agricultural benefit. Further exploration of the chromosomal behavior during meiosis across various hybrids will deepen our understanding of non‐Mendelian inheritance patterns and their implications in plant breeding.

## Core Ideas

- Interspecific hybridization is one of the main mechanisms of plant evolution and speciation.
- In newly emerged hybrids, chromosomes of one species are often eliminated or substituted by those of the other.
- In *Allium cepa × A. roylei* hybrids, *A. roylei* genome is dominant over the *A. cepa* genome.
- This dominance can be reverted by the different proportions of the parental genomes.

**Abbreviations**
BC_1_
: first backcross generation
BC_1_F_1_
: progeny after intercross of the first backcross generation
F_1_
: the first filial generation
gDNA: genomic DNA
GISH: genomic in situ hybridization

## 1 INTRODUCTION

Merging of two distinct genomes via interspecific hybridization opens an arena for their interactions, which, in essence, can be viewed as a battle for survival. It was frequently observed that one of the genomes becomes dominant and overcomes the other one in many ways, including elimination of its chromosomes, which may or may not be achieved by their replacement by chromosomes of the dominant genome (Glombik et al., 2020). This phenomenon is called genome dominance (at chromosome level) and represents another case of non‐Mendelian inheritance. The mechanism responsible for this phenomenon has been an enigma for a long time, but recently, there are more and more indications that it is caused by the differential behavior of the parental chromosomes during meiosis (Svačina et al., 2020). In female meiosis, such behavior is expected: The products of the first meiotic division are transmitted either to the future egg cell (participating in the next generation) or to the polar body (which is eventually eliminated). The difference in the transmission rate of parental chromosomes into either egg cell or to polar body has been reported in hybrid mice (Akera et al., 2017). This phenomenon is called female meiotic drive (Sandler et al., 1959) and was also observed in various plant interspecific hybrids including xFestulolium (*Festuca × Lolium*; Majka et al., 2023), some lily hybrids (Karlov et al., 1999; Khan et al., 2009), *Alstroemeria aurea × A. inodora* (Kamstra et al., 1999), and *Gasteria lutzii × Aloe aristata* (Takahashi et al., 1997). It is so far unclear what causes the differential transmission of the parental chromosomes into the egg cell; however, it has to rely to some extent on a different centromere structure between chromosomes of the two species. Different structures of the centromeres and kinetochores, which mediate the connection of the chromosomes to the meiotic spindle apparatus during cell divisions, are also responsible for non‐Mendelian inheritance of the parental chromosomes during male meiosis, at least in some hybrids. We observed that xFestulolium hybrids display shift in genome composition in favor of *Lolium* genome, and this shift is processed via both female and male meioses (Majka et al., 2023). In male meiosis, univalents (unpaired chromosomes during meiotic division) of *Lolium* are attached to the microtubules of the spindle more frequently than those of *Festuca* and are carried toward the poles. On the contrary, *Festuca* univalents are not attached, linger at the metaphase plate, and form micronuclei, which are the subjects of elimination. This different behavior of univalents was caused by silencing of *Festuca* alleles of two kinetochore genes, namely, *NNF1* and *NDC80* (Majka et al., 2023).

Molecular cytogenetics helped to reveal that interspecific *Allium* hybrids such as *A. cepa × A. roylei* are also subjects of genome dominance. Unlike in the xFestulolium hybrids, male meiosis appears to result in gametes with equal proportion of the parental chromosomes. On the other hand, female gametes appear to favor the chromosomes of the *A. roylei* genome (on average, 2.81 *cepa* chromosomes + 5.14 *roylei* chromosomes), indicating that genome dominance in these hybrids is a consequence of the female meiotic drive (Kopecký et al., 2022). Interestingly, this female meiotic drive does not seem to have the same effect across the entire genome. Some chromosomes of *A. cepa* tend to be replaced by their *A. roylei* counterparts more frequently than others (Kopecký et al., 2022). It also appears that genome dominance is chromosome specific in other hybrids. We have shown that in allotetraploid xFestulolium *F. pratensis* chromosome 5 is replaced by its *L. multiflorum* homoeologue more frequently than any other chromosome (Kopecký et al., 2019). Similarly, Lopez‐Lavalle and Brubaker (2007) observed preferential elimination of some chromosomes and higher transmission of others in *Gossypium hirsutum* × *G. sturtianum* and *G. hirsutum* × *G. australe* hybrids.

Molecular mechanisms of genome dominance and its chromosome specificity, as well as any generalities, if any, are yet to be revealed in different hybrids. Besides that, another intriguing question remains to be answered: can the dominance of a parental genome involved in female meiotic drive be reversed? In other words, can a dominant parental genome (i.e., whose chromosomes are more frequently transmitted to the egg cell at the expense of chromosomes of the other parental genome) become submissive under some circumstances? Here, we show that in a backcross generation (F_1_ [the first filial generation] hybrid crossed to the submissive parent), a switch of the dominant genome appears to occur. While *A. roylei* genome (R) was dominant over that of *A. cepa* (C) in female meiosis of the F_1_ hybrids (proportion of parental genomes 1:1), it became submissive during female meiosis of the BC_1_ (first backcross generation) hybrids (proportion ∼1R:3C).

## 2 MATERIALS AND METHODS

### 2.1 Plant material

The BC_1_ population, consisting of 23 plants, was made by open pollination of F_1_ hybrids of *Allium cepa* ‘Jumbo’ ♀ × *A. roylei* ‘C502’ ♂ with *A. cepa* ‘Jumbo’ pollen in the greenhouse conditions of Wageningen University & Research, the Netherlands. The BC_1_F_1_ (progeny after intercross of the first backcross generation) populations were generated by intercrossing (open pollination) all 23 plants of the BC_1_ population in the experimental field of the Institute of Experimental Botany ASCR in Olomouc, Czech Republic. Only 14 out of the 23 plants produced seeds. Each BC_1_F_1_ population is represented by the progeny of one mother plant pollinated by the pollen of the remaining BC_1_ plants. All 23 and 236 plants of BC_1_ and BC_1_F_1_ populations, respectively, were analyzed by the genomic in situ hybridization (GISH) to determine their chromosome constitutions. All F_1_ plants used for the development of BC_1_ and, subsequently, BC_1_F_1_ populations shared the same parental lineages and came from the original set of van Heusden et al. (2000).

### 2.2 Seed set and germination rate

The number of seeds produced by each of the 23 plants of the BC_1_ population was recorded. Seeds were germinated on wet filter paper, and the germination rate was determined for each plant after 20 days (Table 1).

**Core Ideas**

- Interspecific hybridization is one of the main mechanisms of plant evolution and speciation.
- In newly emerged hybrids, chromosomes of one species are often eliminated or substituted by those of the other.
- In *Allium cepa × A. roylei* hybrids, *A. roylei* genome is dominant over the *A. cepa* genome.
- This dominance can be reverted by the different proportions of the parental genomes.

**TABLE 1:** Genomic composition of individual BC_1_ (first backcross generation) plants, number of seeds obtained after polycross, and germination rate of the seeds.

Mother plant	Number of chromosomes		Number of seeds	Germination rate (%)
	*A. cepa*	*A. roylei*		
**186/15**	14	2	49	63.3
**186/12**	13	3	101	65.3
**186/18**	13	3	65	24.6
**186/20**	13	3	93	66.7
**187/3**	13	3	160	48.2
186/2	12	4	87	30.9
186/9	12	4	0	–
186/11	12	4	0	–
**186/13**	12	4	67	25.0
**186/17**	12	4	76	42.2
186/19	12	4	0	–
**187/4**	11	5	120	25.0
**186/3**	11	5	127	42.2
186/5	11	5	20	25.0
186/14	11	5	0	–
**186/16**	11	5	99	48.5
**186/1**	10	6	49	28.6
**186/7**	10	6	147	15.9
**187/2**	10	6	56	19.6
186/6	9	7	0	–
**186/10**	9	7	145	13.1
188/1	11	5	0	–
42/1	11	5	0	–

*Note*: Mother plants used for the BC_1_F_1_ (progeny after intercross of the first backcross generation) populations production are in bold.

### 2.3 Genomic in situ hybridization, genomic composition analyses, and statistical treatments

Roots of individual plants were collected into iced distilled water for 28 h and fixed in the Carnoy's solution (absolute ethanol/glacial acetic acid, 3:1 v/v). Chromosome preparations from root tips were made according to Masoudi‐Nejad et al. (2002). GISH analyses were done according to Ferreira et al. (2021). Total genomic DNA (gDNA) of *A. cepa* was used as blocking DNA, and total gDNA of *A. roylei* was labeled with digoxigenin (DIG) using the DIG‐Nick Translation Kit (Roche Applied Science) according to the manufacturer's instructions and used as a probe. The probe/blocking DNA ratio was ∼1:150. Signal detection was made with anti‐DIG‐fluorescein isothiocyanate (FITC) conjugate (Roche Applied Science). Chromosomes were counterstained with DAPI (4 6‐Diamidino‐2‐phenylindole) in Vectashield (Vector Laboratories, Oberkochen, Germany). Observations were done using an Olympus AX70 microscope equipped with epifluorescence and a SensiCam B/W camera. Images were captured with Micro Image and processed with Adobe Photoshop v.6 software (Adobe Systems Corporation). As homoeologous recombination does take place in these hybrids, the identity of a chromosome was based on the origin of its centromere—the chromosome was counted as *A. roylei* origin when its (peri)centromeric region was labeled with the *A. roylei* probe, even though the rest of the chromosome might have been of *A. cepa* chromatin (not showing the signal of *A. roylei* probe). This approach was used in several other studies (Kopecký et al., 2022; Majka et al., 2023).

### 2.4 Statistical analyses

We tested the hypothesis that there are no differences between the predicted and observed numbers of *A. cepa* and *A. roylei* chromosomes in the BC_1_F_1_ populations by two alternative procedures. The predicted genome composition (number of *A. cepa* and *A. roylei* chromosomes) of each BC_1_F_1_ population was calculated as a sum of the number of *A. cepa* and *A. roylei* chromosomes present in individual mother plants and the estimated number of *A. cepa* and *A. roylei* chromosomes of the pollinators. The number of seeds obtained from each BC_1_ plant and the genome composition of the individual plants used in the polycross of the BC_1_ plants were used to estimate the genome composition of the pollinators.

First, we used a one‐sample *t*‐test to test the null hypothesis that the difference between the observed and predicted number of *A. cepa* chromosomes in the genome composition of BC_1_F_1_ populations is zero (i.e., predicted numbers = observed numbers). We used this approach because all BC_1_F_1_ plants had an identical number of chromosomes (2*n* = 16), and thus, the contribution of the other parental genome is a redundant parameter in statistical tests used here. The *t*‐test was calculated separately for each comparison (each BC_1_F_1_ population). Second, we used a meta‐analysis of proportions, where the observed and predicted number of chromosomes of *A. cepa* and *A. roylei* within each individual of the BC_1_F_1_ were considered as variables (counts) and each comparison (mother) as a group. Two tests were calculated. The directional zero‐effect test tested the null hypothesis that there is no difference between the predicted and observed chromosome numbers of *A. cepa and A. roylei* in the BC_1_F_1_ generation versus the alternative that there is the same shift of observed versus predicted chromosome numbers (i.e., a nonzero effect). This test was applied for each comparison and over all comparisons (“Combined”). The effect‐equality (heterogeneity) test, using Cochran's *Q*, tested the null hypothesis that variation in genome composition across comparisons is not larger than within comparisons (i.e., homogeneity) versus the alternative that there is larger variation across comparisons rather than within comparisons (i.e., heterogeneity). All statistical analyses were done in NCSS (www.ncss.com).

## 3 RESULTS

**FIGURE 1:**
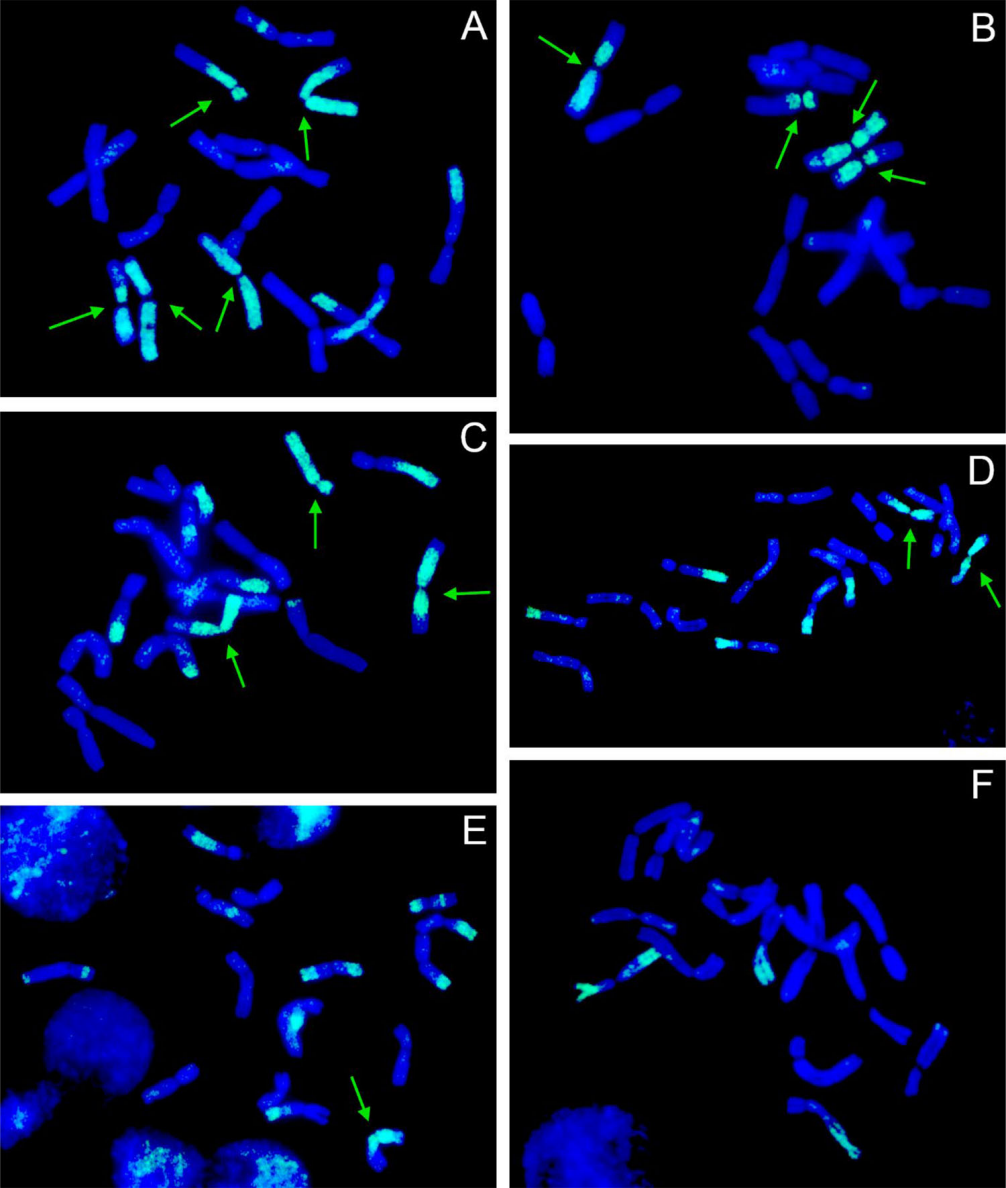
Molecular cytogenetic analysis of *A. cepa × A. roylei* hybrids. Mitotic cells of BC_1_ (A) and BC_1_F_1_ (B–F) plants after genomic in situ hybridization. The total genomic DNA (gDNA) of *A. roylei* was labeled with digoxigenin (green color), and sheared DNA of *A. cepa* was used as blocking DNA (blue color). Note the large variation in the number of *A. roylei* chromosomes (green arrows).

All 23 plants of the BC_1_ population were euploid with 16 chromosomes, and their genome compositions ranged from 14 *A. cepa* chromosomes + 2 *A. roylei* chromosomes (14C + 2R) to 9C + 7R (Figure 1A) with a mean of 11.43C + 4.57R. With eight chromosomes of *A. cepa* delivered through the pollen (the pollinator of F_1_ hybrids), the genome composition of egg cells ranged from 6C + 2R to 1C + 7R. It has to be mentioned that all BC_1_ plants (as well as BC_1_F_1_ plants described below) had homoeologous translocations, and the identity of the chromosome was based on the fluorescent signal spanning (peri)centromeric region of the particular chromosome. These homoeologous recombinations seem to have no effect on the chromosome pairing and segregation (de Vries et al., 1992; Kopecký et al., 2022).

**FIGURE 2:**
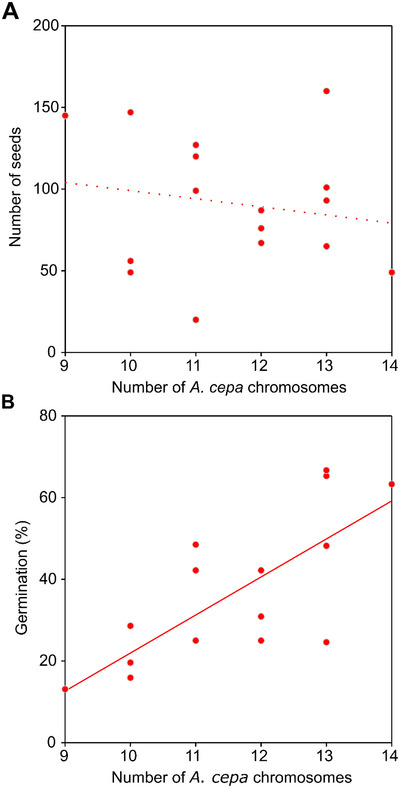
Relationships between genome composition and fertility traits. (A) Nonsignificant correlation between the number of *A. cepa* chromosomes in the mother plants and the number of seeds. (B) Significant correlation between the number of *A. cepa* chromosomes in the mother plants and the germination rate of the seeds obtained from the mother plants after polycross.

All these 23 plants were grown in the experimental field of the Institute of Experimental Botany ASCR to produce the BC_1_F_1_ generation. Of those 23 plants of BC_1_ generation, only 16 plants produced seeds, with different quantities (from 20 to 160; Table 1). There was no correlation between the proportion of parental genomes (number of *A. cepa* chromosomes) and the number of seeds produced (Pearson's *r* = −0.174, *p* = 0.519; Figure 2A). All seeds were sown on Petri dishes, and germinated seeds were counted after 20 days. Germination rates ranged from 13.1% to 66.7% (Table 1). In general, plants with a higher proportion of the *A. cepa* chromosomes had a higher germination rate compared to those with fewer *A. cepa* chromosomes; however, the correlation was not statistically significant (Pearson's *r* = 0.744, *p* = 0.001; Figure 2B).

The number of seeds produced (as a proxy of fertility/pollen contribution of individual plants) and the genome composition of individual BC_1_ plants used in this polycross were used to estimate the genome composition of the pollinators. Such predicted numbers of chromosomes (11.42C + 4.58R) are almost the same as the ones when the number of seeds is not considered (11.48C + 4.52R). Using this approximation for the pollinators and the exact genome composition of the maternal plants, we were able to predict mean genome composition of the progeny of each maternal plant. The predicted genome composition of each of the BC_1_F_1_ populations (progeny of one mother plant) was compared with the averaged genome composition of all plants of each BC_1_F_1_ family (from 3 to 57 plants) revealed by GISH (Figure 1B–D). The genome composition of 236 BC_1_F_1_ plants ranged from 8C + 8R to 16C + 0R with the mean of 13.39C + 2.61R (Table 2). Thus, the number of *A. roylei* chromosomes in BC_1_F_1_ is statistically significantly lower than that in BC_1_.

**TABLE 2:** Descriptive statistics of comparisons between predicted and observed genome composition of 14 BC_1_F_1_ (progeny after intercross of the first backcross generation) populations. Both observed and predicted chromosome numbers of *A. cepa* and *A. roylei* in the BC_1_F_1_ generation are presented. Observed numbers are represented by mean and standard deviation (SD) of each BC_1_F_1_ population. One‐sample *t*‐test tests the null hypothesis that the difference of observed and predicted number of *A. cepa* chromosomes in genome composition of BC_1_F_1_ populations is zero (i.e., predicted numbers = observed numbers). The *t*‐tests were calculated separately for each BC_1_F_1_ population. The directional zero‐effect test tests the null hypothesis that there is no difference between predicted and observed chromosome numbers of *A. cepa* and *A. roylei* in the BC_1_F_1_ generation versus the alternative (the same shift of observed versus predicted chromosome numbers, i.e., nonzero effect) for both within each comparison and over all comparisons (“Combined”).

Mother plant (number of progeny plants BC_1_F_1_)	Observed chromosome numbers		Predicted chromosome numbers		One‐sample *t*‐test			Directional zero‐effect test		
	*A. cepa* mean (SD)	*A. roylei* mean	*A. cepa*	*A. roylei*	*T*	*df*	*p*	Chi‐square^a^	*df*	*p*
186/15 (35)	14.37 (1.15)	1.63	12.71	3.29	8.6670	35	**<0.0001**	15.6963	1	**0.0001**
186/12 (57)	13.88 (1.27)	2.12	12.21	3.79	9.9730	57	**<0.0001**	22.5188	1	**<0.0001**
186/18 (10)	14.40 (0.80)	1.60	12.21	3.79	9.0697	10	**<0.0001**	8.6100	1	**0.0033**
186/20 (17)	13.35 (0.97)	2.65	12.21	3.79	4.7202	16	**0.0002**	3.5029	1	**0.0613**
187/3 (44)	13.34 (1.36)	2.66	12.21	3.79	5.4250	43	**<0.0001**	7.8918	1	**0.0050**
186/13 (3)	13.33 (0.47)	2.67	11.71	4.29	4.8631	2	**0.0398**	1.2606	1	0.2615
186/17 (8)	14.00 (0.50)	2.00	11.71	4.29	12.1054	7	**<0.0001**	6.9256	1	**0.0085**
187/4 (7)	13.14 (1.73)	2.86	11.21	4.79	2.7396	6	**0.0338**	3.1992	1	0.0737
186/3 (7)	13.86 (1.12)	2.14	11.21	4.79	5.7594	6	**0.0012**	6.1675	1	**0.0130**
186/16 (8)	14.12 (1.45)	1.88	11.21	4.79	5.3060	7	**0.0011**	8.7168	1	**0.0032**
186/1 (10)	11.80 (1.72)	4.20	10.71	5.29	2.0968	10	0.0624	1.3435	1	0.2464
186/7 (7)	11.00 (2.3)	5.00	10.71	5.29	0.3107	6	0.7665	0.0239	1	0.8772
187/2 (5)	13.40 (1.20)	2.60	10.71	5.29	4.4795	4	**0.0110**	4.8799	1	**0.0272**
186/10 (18)	10.61 (1.77)	5.39	10.21	5.79	0.9830	18	0.3386	0.3436	1	0.5577
Combined	–	–	–	–	–	–	–	73.4090	1	**<0.0001**

*Note*: Significant *p* values (*p* ≤ 0.05) are in bold.

Abbreviation: *df*, degrees of freedom.

^a^ The chi‐square value tests the null hypothesis that all effects are zero versus the alternative that all studies had the same, nonzero effect.

**FIGURE 3:**
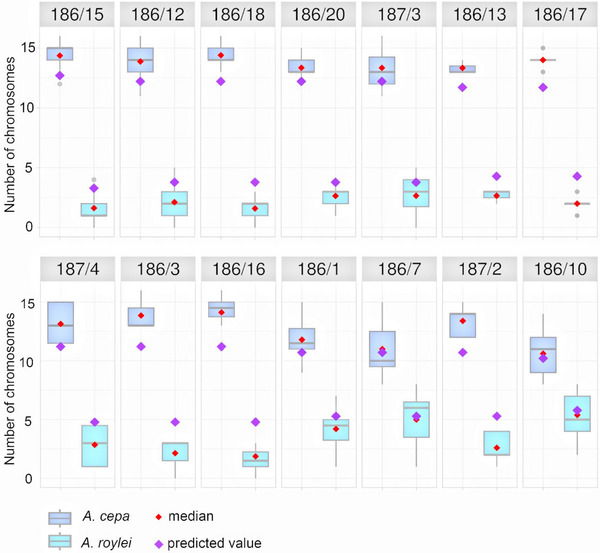
Predicted and observed numbers of chromosomes in BC_1_F_1_ populations *A. cepa × A. roylei* hybrids. Comparison of the predicted (violet diamonds) and observed (red diamond represents median, box plot displays individual quartiles, and the thick line represents mean) numbers of *A. cepa* and *A. roylei* chromosomes. Note that in all cases, the predicted numbers of *A. cepa* chromosomes are always lower than the observed numbers, even though in three populations (186/1, 186/7, and 186/10), the differences are statistically nonsignificant.

We made 14 comparisons of the predicted and observed genome compositions of the BC_1_F_1_ populations (Table 2, Figure 3). All BC_1_F_1_ plants were euploid with 16 chromosomes. In 11 out of 14 comparisons (i.e., per maternal plants), the one‐sample *t*‐tests showed that the predicted and observed genome compositions differed; mean numbers of the observed *A. cepa* chromosomes were significantly higher than those predicted (Table 2). In the remaining three comparisons (from mother plants 186/1, 186/7, and 186/10), while mean observed numbers of *A. cepa* chromosomes did not statistically significantly differ from the predicted ones, the trend was the same as in the other comparisons (i.e., the observed number of *A. cepa* chromosomes was higher than the predicted number; Table 2, Figure 3). Overall, on average, there was an excess of about 1.7 *A. cepa* chromosomes over the value predicted for the BC_1_F_1_ population (Table 2). There were differences between individual families of the BC_1_F_1_ population; progenies of the BC_1_ plants with more *A. roylei* chromosomes tended to retain those chromosomes more efficiently than those having less *A. roylei* chromosomes (Figure 3). This indicates that more equal proportion of the parental chromosomes was associated with higher stability of such proportion.

Using the alternative meta‐analytical approach, the mean (95% confidence interval) odds ratio of observed and predicted numbers of chromosomes of *A. cepa* and *A. roylei* presenting combined estimates for all comparisons (mothers) was 1.6140 (1.4466, 1.8008). This suggests that, in general, the number of observed *A. cepa* and *A. roylei* chromosomes within genome composition of “Combined” BC_1_F_1_ population differs from those predicted. The results of the overall null hypothesis testing, using the directional zero‐effect test, showed the same nonzero effect in different comparisons (BC_1_F_1_ populations). Consequently, the effect‐equality (heterogeneity) test showed that the variation in genome composition across comparisons is not larger than within comparisons (Cochran's *Q* = 108.8386, *df* = 239, *p* = 1.0000). Our both approaches strongly suggest that the *A. cepa* genome is more dominant in BC_1_F_1_ plants (i.e., BC_1_F_1_ populations have more *A. cepa* chromosomes than predicted). This pattern is common to all comparisons within the entire BC_1_F_1_ population studied.

## 4 DISCUSSION

The utilization of interspecific hybrids in plant breeding and agriculture depends heavily on the stability of their genomes. The main issue in allopolyploids, where each chromosome has its own homologous partner to pair with, is prevention of homoeologous pairing resulting in regular, diploid‐like meiosis (Svačina et al., 2020). Such stability is usually achieved by some strict criteria for crossover, hence, chiasmata formation. Tetraploid and hexaploid wheats (*Triticum* spp.) are the perfect example of the perfect meiotic stability of allopolyploids, due to the presence of the meiotic regulator *Ph1* (*pairing homoeologous 1*; Sears & Okamoto, 1958). However, this is not possible in homoploid (diploid) hybrids where only one set of chromosomes from each parental species is present. Not surprisingly, most homoploids are sterile as their constitutive genomes are unable to pair in metaphase I of meiosis, and no functional gametes are formed. Interestingly, there are some homoploid hybrids, as well as some allopolyploids, where homoeologous chromosome pairing takes place (Svačina et al., 2020). Such homoeologous chromosome pairing creates an opportunity for the competition of parental chromosomes during meiotic division, gametogenesis, and perhaps competition for fertilization (Glombik et al., 2020). Various hybrids with homoeologous chromosome pairing often show the so‐called female meiotic drive. This phenomenon is created when chromosomes of the “dominant” genome are more frequently transmitted to the dyad after the first meiotic division, which eventually will produce the egg; chromosomes of the “submissive” genome are more frequently transmitted to the polar body (and thus, not contributing to the next generation). This has been observed in hybrids of lily, xFestulolium, and *A. cepa × A. roylei* (Karlov et al., 1999; Khan et al., 2009; Kopecký et al., 2009, 2005, 2022; Majka et al., 2023). What exactly is behind this phenomenon is still an open question in plant hybrids, but this article does not intend to address it. Here, we aimed to explore the possibility to manipulate (reverse) genome dominance at the chromosome level.

During female meiosis of hybrids of *A. cepa × A. roylei*, chromosomes of *A. roylei* are transmitted to egg cells more frequently than those of *A. cepa*. This shifts the genome composition in favor of *A. roylei* chromosomes. In other words, chromosomes of *A. cepa* are substituted by those of *A. roylei*. Male meiosis is symmetrical, so no shift in the proportion of parental chromosomes takes place. Here, we demonstrate that altered starting proportions of the chromosomes from parental genomes can affect the direction of genome dominance. *A. roylei* genome, dominant during the meiotic division of the F_1_ hybrids with equal proportions of parental genomes—14 *A. roylei* + 14 *A. cepa* chromosomes—becomes submissive during meiosis of BC_1_ population where, after a backcross to *A. cepa*, the proportion of its chromosomes became substantially lower. We are not aware of any report of a similar situation in other interspecific hybrids. However, some indications of the effect of the proportion of parental genomes on the identity of the dominant genome can be extracted from studies of the genome (in)stability of newly developed hybrids and allopolyploids such as synthetic *Brassica* hybrids and xFestulolium hybrids. There are three genomes in *Brassica*, A genome of *B. rapa*, B genome of *B. nigra*, and C genome of *B. oleracea*, forming three allotetraploids *B. juncea* (AABB), *B. napus* (AACC), and *B. carinata* (BBCC). Data extracted from Katche et al. (2023) illustrate that the C‐genome chromosomes were lost much more frequently in allopolyploids BBAC compared to AABC (32.8 vs. 18.4%). On the other hand, elimination of the A‐genome chromosomes was about the same in BBAC and CCAB allopolyploids (16.9 vs. 16.0%). A study by Leflon et al. (2006) about the genome composition of the progeny of reciprocally backcrossed and intercrossed triploid AAC hybrids suggested another feature: there are differences in the transmission of chromosomes between male and female meiosis. In female meiosis, the transmission of chromosomes seems to copy the binomial distribution (random transmission); male meiosis displays higher transmission rate of the chromosomes. This is the same pattern we observed here, both in *A. cepa × A. roylei* and xFestulolium hybrids, where male and female meiosis differ in the transmission rates of parental chromosomes (Kopecký et al., 2022; Majka et al., 2023).

In xFestulolium, King et al. (1999) and Kosmala et al. (2006) found higher numbers of *Festuca* univalents compared to those of *Lolium* in triploid LpLpFp and FpLmLm hybrids (3.32 vs. 0.54 and 2.24 vs. 0.71, respectively). This indicates that while pairing of homoeologues was frequent, it was not as frequent as that of homologues and that the rate of homoeologue pairing depends on the phylogenetic relationships of the parental genomes (*F. pratensis* appears more closely related to *L. multiflorum* than to *L. perenne*; reviewed in Kopecký et al., 2008). However, using the number of univalents in this hybrid is somewhat problematic, as Majka et al. (2023) found that while the majority of *Festuca* univalents were lagging during male meiosis and later were micronucleated and eliminated, the majority of *Lolium* univalent were attached to the karyokinetic spindles and were transmitted to the developing gametes. Studies on xFestulolium with different proportion of parental genomes revealed another interesting feature: while introgression cultivars (with very few chromosomes or chromosome segments of *F. pratensis* in the *Lolium* background) tend to heavily eliminate *Festuca* chromatin, amphiploid cultivars with the genomic proportion 2:1 to 3:1 (in a favor of *Lolium*) were relatively stable, and there was no further shift toward *Lolium* genome. It was hypothesized that this is due to hybrid vigor favoring plants with this proportion of parental genomes over the genotypes with a more unequal proportion (Kopecký et al., 2019, 2017).

Besides the transmission of chromosomes of the parental genomes, the proportion of parental genomes also appears to affect hybrid fertility. Triploid xFestulolium hybrids with two *L. multiflorum* genomes are fertile, while those with two *F. pratensis* genomes are mostly sterile (Humphreys & Thorogood, 1993; Jauhar, 1993; Naganowska et al., 2001). This is similar to the AAC and ACC triploid hybrids in *Brassica*. While the former ones were largely fertile, the latter ones were completely sterile (Cao et al., 2023; Yang et al., 2017). This is in contrast to our observations on *Allium* hybrids in this study. Our results indicate that the proportion of parental genomes has only marginal, if any, effect on the fertility of the hybrids. We obtained no seed from one plant with the constitution of 9C + 7R chromosomes, while another plant with such constitution gave as many as 145 seeds. Similarly, three plants with the constitution of 12C + 4R were sterile, while other three produced 87, 76, and 67 seeds (Table 1). Despite the same proportion of the parental genomes, it is likely that the chromosome constitution differs in these plants. As we did not identify individual chromosomes present but only assigned them to parental genomes by GISH, we are lacking the information which individual chromosomes (or chromosome regions) are in double dose, single dose, or missing completely. It seems that some chromosomes or chromosome regions are more prone to transmission (or elimination) than others, and we could study this in more detail using single nucleotide polymorphism markers that have been obtained for this cross (Kopecký et al., 2022; Scholten et al., 2016). For example, two regions on *A. cepa* chromosomes 5 and 8 are transmitted at a frequency exceeding 50% in *A. cepa × A. roylei* hybrids, while other parts of *A. cepa* genome show the transmission rate below 50% (Kopecký et al., 2022). Allotetraploid *Brassica* (genome composition BBAC) also display preferential retention of chromosomes (A04, A05, A06, and A08) and preferential elimination of others (C4 and C5; Katche et al., 2023). Similarly, chromosome 5F is more frequently eliminated in substitution lines of *L. multiflorum × F. pratensis* than any other chromosome (Kopecký et al., 2019).

The results from all three models provide some overview on the effect of proportion of parental genomes on the genome dominance and fertility. It seems that the transmission of the chromosomes of the parental genomes is affected by the proportion of these genomes in all hybrids. However, the extent varies significantly, and the predictions on the transmission of the chromosomes of the parental genomes cannot be extrapolated from the studies of different hybrids. However, the results of *L. multiflorum × F. pratensis* and *L. perenne × F. pratensis* indicates that it can be, to some extent, estimated from closely related hybrids. Moreover, it is evident that specific characteristics, such as lethal factors, hybrid vigor, or silencing of species‐specific kinetochore genes, may affect various features from chromosome pairing and transmission of the chromosomes to lethality of the hybrids.

## AUTHOR CONTRIBUTIONS

**David Kopecký**: Conceptualization; data curation; formal analysis; funding acquisition; investigation; methodology; project administration; resources; software; supervision; validation; visualization; writing—original draft. **Martin Duchoslav**: Investigation; methodology; visualization; writing—review and editing. **Olga Scholten**: Resources; writing—review and editing. **Jana Kneřová**: Investigation; visualization; writing—review and editing. **Marek Szecówka**: Investigation; visualization; writing—review and editing.

## CONFLICT OF INTEREST STATEMENT

The authors declare no conflicts of interest.

## Data Availability

All data generated or analyzed during this study are included in this article.

## References

[tpg270016-bib-0001] Akera, T. , Chmátal, L. , Trimm, E. , Yang, K. , Aonbangkhen, C. , Chenoweth, D. M. , Janke, C. , Schultz, R. M. , & Lampson, M. A. (2017). Spindle asymmetry drives non‐Mendelian chromosome segregation. Science, 358, 668–672. 10.1126/science.aan0092 29097549 PMC5906099

[tpg270016-bib-0002] Cao, Y. , Zhao, K. , Xu, J. , Wu, L. , Hao, F. , Sun, M. , Dong, J. , Chao, G. , Zhang, H. , Gong, X. , Chen, Y. , Chen, C. , Qian, W. , Pires, J. , Edger, P. , & Xiong, Z. (2023). Genome balance and dosage effect drive allopolyploid formation in *Brassica* . Proceedings of The National Academy of Sciences of the United States of America, 120, Article e2217672120. 36989303 10.1073/pnas.2217672120 PMC10083598

[tpg270016-bib-0003] de Vries, J. N. , Wietsma, W. A. , & de Vries, T. (1992). Introgression of leaf‐blight resistance from *Allium roylei* Stearn into onion (*Allium cepa* L.). Euphytica, 62, 127–133.

[tpg270016-bib-0031] Ferreira, M. T. M. , Glombik, M. , Perničková, K. , Duchoslav, M. , Scholten, O. , Karafiátová, M. , Techio, V. H. , Doležel, J. , Lukaszewski, A. J. , & Kopecký, D. (2020). Direct evidence for crossover and chromatid interference in meiosis of two plant hybrids *(Lolium multiflorum×Festuca pratensis* and *Allium cepa×A. roylei)* . Journal of Experimental Botany, 72(2), 254–267. 10.1093/jxb/eraa455 PMC7853598 33029645

[tpg270016-bib-0004] Glombik, M. , Bačovský, V. , Hobza, R. , & Kopecký, D. (2020). Competition of parental genomes in plant hybrids. Frontiers in Plant Science, 11, Article 200. 10.3389/fpls.2020.00200 32158461 PMC7052263

[tpg270016-bib-0005] Humphreys, M. , & Thorogood, D. (1993). Disturbed Mendelian segregation at isozyme marker loci in early backcrosses of *Lolium multiflorum* × *Festuca pratensis* hybrids to *L. multiflorum* . Euphytica, 66, 11–18. 10.1007/BF00023503

[tpg270016-bib-0006] Jauhar, P. P. (1993). Cytogenetics of the Festuca‐Lolium complex: Relevance to breeding. Springer.

[tpg270016-bib-0007] Kamstra, S. A. , Kuipers, A. G. J. , De Jeu, M. J. , Ramanna, M. S. , & Jacobsen, E. (1999). The extent and position of homoeologous recombination in a distant hybrid of *Alstroemeria*: A molecular cytogenetic assessment of first generation backcross progenies. Chromosoma, 108, 52–63. 10199956 10.1007/s004120050351

[tpg270016-bib-0008] Karlov, G. I. , Khrustaleva, L. I. , Lim, K. B. , & van Tuyl, J. M. (1999). Homoeologous recombination in 2n‐gametes producing interspecific hybrids of *Lilium* (Liliaceae) studied by genomic in situ hybridization (GISH). Genome, 42, 681–686. 10.1139/g98-167

[tpg270016-bib-0009] Katche, E. , Schierholt, A. , Becker, H. , Batley, J. , & Mason, A. (2023). Fertility, genome stability, and homozygosity in a diverse set of resynthesized rapeseed lines. Crop Journal, 11, 468–477. 10.1016/j.cj.2022.07.022

[tpg270016-bib-0010] Khan, N. , Barba‐Gonzalez, R. , Ramanna, M. S. , Visser, R. G. F. , & Van Tuyl, J. M. (2009). Construction of chromosomal recombination maps of three genomes of lilies (*Lilium*) based on GISH analysis. Genome, 52, 238–251. 10.1139/G08-122 19234552

[tpg270016-bib-0011] King, I. , Morgan, W. , Harper, J. , & Thomas, H. (1999). Introgression mapping in the grasses. II. Meiotic analysis of the *Lolium perenne*/*Festuca pratensis* triploid hybrid. Heredity, 82, 107–112. 10.1038/sj.hdy.6884680

[tpg270016-bib-0012] Kopecký, D. , Bartoš, J. , Zwierzykowski, Z. , & Doležel, J. (2009). Chromosome pairing of individual genomes in tall fescue (*Festuca arundinacea* Schreb.), its progenitors, and hybrids with Italian ryegrass (*Lolium multiflorum* Lam.). Cytogenetic and Genome Research, 124, 170–178. 10.1159/000207525 19420930

[tpg270016-bib-0013] Kopecký, D. , Horáková, L. , Duchoslav, M. , & Doležel, J. (2019). Selective elimination of parental chromatin from introgression cultivars of xFestulolium (*Festuca* × *Lolium*). Sustainability, 11, Article 3153. 10.3390/su11113153

[tpg270016-bib-0014] Kopecky, D. , Lukaszewski, A. , & Gibeault, V. (2005). Reduction of ploidy level by androgenesis in intergeneric *Lolium*‐*Festuca* hybrids for turf grass breeding. Crop Science, 45, 274–281. 10.2135/cropsci2005.0274a

[tpg270016-bib-0015] Kopecký, D. , Lukaszewski, A. J. , & Doležel, J. (2008). Cytogenetics of Festulolium (*Festuca* × *Lolium* hybrids). Cytogenetic and Genome Research, 120, 370–383. 10.1159/000121086 18504366

[tpg270016-bib-0016] Kopecký, D. , Scholten, O. , Majka, J. , Burger‐Meijer, K. , Duchoslav, M. , & Bartoš, J. (2022). Genome dominance in *Allium* hybrids (*A. cepa* × *A. roylei*). Frontiers in Plant Science, 13, Article 854127. 10.3389/fpls.2022.854127 35371123 PMC8965639

[tpg270016-bib-0017] Kopecký, D. , Šimoníková, D. , Ghesquière, M. , & Doležel, J. (2017). Stability of genome composition and recombination between homoeologous chromosomes in *Festulolium* (*Festuca* × *Lolium*) cultivars. Cytogenetic and Genome Research, 151, 106–114. 10.1159/000458746 28297695

[tpg270016-bib-0018] Kosmala, A. , Zwierzykowska, E. , & Zwierzykowski, Z. (2006). Chromosome pairing in triploid intergeneric hybrids of *Festuca pratensis* with *Lolium multiflorum*, revealed by GISH. Journal of Applied Genetics, 47, 215–220. 10.1007/BF03194626 16877799

[tpg270016-bib-0019] Leflon, M. , Eber, F. , Letanneur, J. C. , Chelysheva, L. , Coriton, O. , Huteau, V. , Ryder, C. D. , Barker, G. , Jenczewski, E. , & Chèvre, A. M. (2006). Pairing and recombination at meiosis of *Brassica rapa* (AA) × *Brassica napus* (AACC) hybrids. Theoretical and Applied Genetics, 113, 1467–1480. 10.1007/s00122-006-0393-0 16983552

[tpg270016-bib-0020] Lopez‐Lavalle, L. A. B. , & Brubaker, C. L. (2007). Frequency and fidelity of alien chromosome transmission in *Gossypium* hexaploid bridging populations. Genome, 50, 479–491. 17612617 10.1139/g07-030

[tpg270016-bib-0021] Majka, J. , Glombik, M. , Doležalová, A. , Kneřová, J. , Ferreira, M. T. M. , Zwierzykowski, Z. , Duchoslav, M. , Studer, B. , Doležel, J. , Bartoš, J. , & Kopecký, D. (2023). Both male and female meiosis contribute to non‐Mendelian inheritance of parental chromosomes in interspecific plant hybrids (*Lolium* × *Festuca*). New Phytologist, 238, 624–636. 10.1111/nph.18753 36658468

[tpg270016-bib-0022] Masoudi‐Nejad, A. , Nasuda, S. , Mcintosh, R. A. , Endo, T. R. , & Endo, T. R. (2002). Transfer of rye chromosome segments to wheat by a gametocidal system. Chromosome Research, 10, 349–357. 10.1023/A:1016845200960 12296517

[tpg270016-bib-0023] Naganowska, B. , Zwierzykowski, Z. , & Zwierzykowska, E. (2001). Meiosis and fertility of reciprocal triploid hybrids of *Lolium multiflorum* with *Festuca pratensis* . Journal of Applied Genetics, 42, 247–255. 14564031

[tpg270016-bib-0024] Sandler, L. , Hiraizumi, Y. , & Sandler, I. (1959). Meiotic drive in natural populations of *Drosophila melanogaster*. 1. The cytogenetic basis of segregation distortion. Genetics, 44, 233–250. 10.1093/genetics/44.2.233 17247821 PMC1209945

[tpg270016-bib-0025] Scholten, O. E. , van Kaauwen, M. P. W. , Shahin, A. , Hendrickx, P. M. , Keizer, L. C. P. , Burger, K. , van Heusden, A. W. , van der Linden, C. G. , & Vosman, B. (2016). SNP‐markers in *Allium* species to facilitate introgression breeding in onion. BMC Plant Biology, 16, Article 187. 10.1186/s12870-016-0879-0 27576474 PMC5006257

[tpg270016-bib-0026] Sears, E. R. , & Okamoto, M. (1958). Intergenomic chromosome relationship in hexaploid wheat. In Proceedings of 10th international congress of genetics (pp. 258–259). University of Toronto Press.

[tpg270016-bib-0027] Svačina, R. , Sourdille, P. , Kopecký, D. , & Bartoš, J. (2020). Chromosome pairing in polyploid grasses. Frontiers in Plant Science, 11, Article 1056. 10.3389/fpls.2020.01056 32733528 PMC7363976

[tpg270016-bib-0028] Takahashi, C. , Leitch, I. J. , Ryan, A. , Bennett, M. D. , & Brandham, P. E. (1997). The use of genomic in situ hybridization (GISH) to show transmission of recombinant chromosomes by a partially fertile bigeneric hybrid, *Gasteria lutzii* × *Aloe aristata* (Aloaceae), to its progeny. Chromosoma, 105, 342–348. 9087376

[tpg270016-bib-0029] van Heusden, A. W. , van Ooijen, J. W. , Vrielink‐van Ginkel, R. , Verbeek, W. H. J. , Wietsma, W. A. , & Kik, C. (2000). A genetic map of an interspecific cross in *Allium* based on amplified fragment length polymorphism (AFLP^TM^) markers. Theoretical and Applied Genetics, 100, 118–126.

[tpg270016-bib-0030] Yang, Y. , Wei, X. , Shi, G. , Wei, F. , Braynen, J. , Zhang, J. , Tian, B. , Cao, G. , & Zhang, X. (2017). Molecular and cytological analyses of A and C genomes at meiosis in synthetic allotriploid *Brassica* hybrids (ACC) between *B. napus* (AACC) and *B. oleracea* (CC). Journal of Plant Biology, 60, 181–188. 10.1007/s12374-016-0221-2

